# Exploring the role and functionality of trial steering committees

**DOI:** 10.1186/1745-6215-16-S2-P189

**Published:** 2015-11-16

**Authors:** Nicola Harman, Elizabeth Conroy, Steff Lewis, Gordon Murray, John Norrie, Matthew Sydes, Athene Lane, Doug Altman, Colin Baigent, Judith Bliss, Marion Campbell, Diana Elbourne, Stephen Evans, Peter Sandercock, Carrol Gamble

**Affiliations:** 1 North West Hub for Trials Methodology Research, Liverpool, UK; 2 Edinburgh MRC Hub for Trials Methodology Research, Edinburgh, UK; 3 MRC Clinical Trials Unit at UCL, London, UK; 4 Centre for Healthcare Randomised Trials (CHaRT), Aberdeen, UK; 5 Bristol Randomised Trials Collaboration Trials Unit, Bristol, UK; 6 Clinical Trial Service Unit & Epidemiological Studies Unit, Oxford, UK; 7 Usher Institute of Population Health Sciences and Informatics, Edinburgh, UK; 8 Department of Medical Statistics, London School of Hygiene and Tropical Medicine, London, UK; 9 Centre for Statistics in Medicine, University of Oxford, Oxford, UK; 10 Health Services Research Unit, University of Aberdeen, Aberdeen, UK; 11 School of Clinical Sciences, University of Edinburgh, Edinburgh, UK; 12 Institute of Cancer Research, Clinical Trials & Statistics Unit, London, UK

## Background

Independent oversight of clinical trials, recommended by Good Clinical Practice, is typically provided by an independent advisory Data Monitoring Committee (DMC) and an independent executive committee, to whom the DMC make recommendations. The detailed role and functionality of this executive committee, known as the Trial Steering Committee (TSC) have not been studied since proposed by the MRC.

## Methods

An expert panel was convened comprising statisticians, clinicians and trial methodologists with prior TSC membership experience. Twelve questions about the role and functionality of the TSC were discussed by the panel at two full-day meetings. Each meeting was transcribed in full and discussions summarised.

## Results

The expert panel reached agreement on the role of the TSC, who they worked for, membership and the definition of independence, and the experience and training needed. The management of ethical issues, difficult/complex situations and issues the TSC should not ask the DMC to make recommendations upon were more difficult to discuss without specific examples. Additional topics discussed, not identified by the DAMOCLES study but pertinent to the role of the TSC, included: indemnity; lifespan of the TSC and role of the sponsor.

## Conclusions

Expert panel discussion and subsequent recommendations will contribute to revision and update of the MRC TSC terms of reference. Uncertainty still remains in some areas due to the absence of published real-life examples. A repository for case studies would be useful. Public contribution to trial oversight was not discussed and warrants further consideration.

